# Progress in Therapy Development for Amyotrophic Lateral Sclerosis

**DOI:** 10.1155/2012/187234

**Published:** 2012-07-04

**Authors:** Kalina Venkova-Hristova, Alexandar Christov, Zarine Kamaluddin, Peter Kobalka, Kenneth Hensley

**Affiliations:** ^1^Department of Pathology, University of Toledo Medical Center, 3000 Arlington Avenue, MS1090, Toledo, OH 43614, USA; ^2^Department of Neurosciences, University of Toledo Medical Center, 3000 Arlington Avenue, MS1090, Toledo, OH 43614, USA

## Abstract

Amyotrophic lateral sclerosis (ALS) is a progressive neurodegenerative disease that cannot be slowed substantially using any currently-available clinical tools. Through decades of studying sporadic and familial ALS (SALS and FALS), researchers are coming to understand ALS as a complex syndrome with diverse genetic and environmental etiologies. It is know appreciated that motor neuron degeneration in ALS requires active (gain of function) and passive (loss of function) events to occur in non-neuronal cells, especially astrocytes and microglia. These neuroinflammatory processes produce paracrine factors that detrimentally affect motor neurons, precipitating protein aggregation and compromising cytoskeletal integrity. The result is a loss of neuronal homeostasis and progressive die-back of motor axons culminating in death of the afflicted motor neurons. This review will discuss experimental therapeutics that have been tested in murine ALS models, with an emphasis on those that have progressed to human clinical trials. Reasons will be considered for the frequent failure of preclinical successes to translate into positive clinical outcomes. Finally, this review will explore current trends in experimental therapeutics for ALS with emphasis on the emerging interest in axon guidance signaling pathways as novel targets for pharmacological support of neural cytoskeletal structure and function in order to slow ALS.

## 1. Introduction

Amyotrophic lateral sclerosis (ALS; colloquially referred to as Lou Gehrig's disease in American English and Motor Neurone Disease in British English) is one member of a family of anterior (ventral) horn diseases that cause progressive, irreversible degeneration and ultimately death of spinal motor neurons and their cortical efferents [1]. Other anterior horn diseases include Charcot-Marie-Tooth disease, spinal muscular atrophy, progressive motor atrophy (PMA), poliomyelitis, and West Nile virus. ALS is anatomically distinguished from other anterior horn diseases and motor neuropathologies by involvement of both upper and lower motor tracts with a relative sparing of sensory neural degeneration, though sensory involvement is present in a subset of ALS patients.

ALS is also distinguished from other motor neuron diseases by its frustrating lack of definable genetic causes and generally enigmatic etiology. Approximately, one fifth of ALS cases are hereditary, but even within this subset there are currently thirteen confirmed Mendelian mutations encoding proteins in disparate pathways that appear at first glance to be minimally interconnected (Table 1) ([2]; also see the ALSoD database, http://alsod.iop.kcl.ac.uk/, [3]). It may be significant that most of the Mendelian factors associated with FALS code for proteins involved in cellular mass transport (either axonal transport or vesicle trafficking), or else code for proteins whose malfunction results in macromolecular aggregates that could impede these transport processes. This is a concept that will be explored in more depth later in this paper.

Amongst the remaining majority of sporadic ALS (SALS) cases, there are few confirmed genetic or environmental risk factors. To date, several independent genomewide association studies have been conducted in an effort to identify genetic contributors to SALS but these have resulted in short, nonoverlapping lists of candidates [2, 5–7]. Three genetic loci have been found that confer significant but small increases in SALS risk (Table 1). Thus, definitive diagnosis of SALS relies upon a combination of primary clinical findings and systematic exclusion of other potential genetic and environmental explanations.

The comparative lack of understanding of ALS etiology has understandably hindered effective therapy development. Nonetheless, there has, for decades, been consistent global research effort to find new targets and therapies for slowing ALS. This passionate effort is driven in part by the severity of ALS clinical presentation, the invariably fatal prognosis, and a growing appreciation that ALS shares many biochemical and cell biological commonalities with other much more prevalent neurological diseases. Thus, understanding the essential neurobiology of ALS could open new vistas for treating neurodegeneration in general. The identification of FALS genes and production of robust animal models (particularly of superoxide dismutase-1 (SOD1) transgenic mice in the early 1990s) have massively accelerated the pace of ALS research and therapy development efforts [8–11]. To date, there have been many preclinical drug tests in these mouse models, with numerous published studies demonstrating modest benefits. Unfortunately, there have been very few replicated preclinical studies that succeeded in producing more than 10% life extension in ALS mice, whereas several promising therapies that were successful in murine trials failed in human clinical trials [10].

This paper will discuss the history of ALS therapy development efforts from the perspective of therapy targets, review the outcomes of major clinical trials, and suggest possible pathways for future preclinical and clinical investigation. Emphasis will be placed on discussion of past therapy development efforts that culminated in human trials, and a specific discussion will be undertaken of probably reasons why successful preclinical strategies have often failed to translate into positive outcomes during human clinical trials.

## 2. Targeting Motor Neuron Death Pathways by Blocking Glutamate-Linked Excitotoxicity

The central feature of ALS is death of the motor neurons. Thus, the earliest strategies to combat ALS focused on intrinsic neuron death pathways, and to this day there is much active research in this area. The key questions are why do the motor neurons die and what pathways might be pharmacologically manipulated for therapeutic benefit? Even prior to the identification of SOD1 mutations in FALS, it was appreciated that human ALS nervous system tissue exhibited protein aggregation, oxidative stress, and loss of cellular homeostasis related to defective glutamate transport [12]. This cytological picture was corroborated when the first SOD1^G93A^ and SOD1^G85R^ transgenic mouse models were introduced in the mid-1990s. The involvement of SOD1 implied, at the time, a likely redox component to disease mapping directly to toxic SOD1 gains-of-function [8–10, 13]. Moreover, the mice were found to suffer a loss in the glial glutamate transporter EAAT2 [13]. By this time in history, it was understood that excess extracellular glutamate could trigger harmful Ca^2+^ influx leading to downstream neurotoxicity, including loss of mitochondrial functionality and concomitant oxidative stress. It was therefore not surprising that some of the first experimental treatments applied to these transgenic models involved administering antioxidants such as vitamin E (alpha tocopherol), alone or in combination with the NMDA (glutamate) receptor antagonists [8, 9]. 

These early studies appeared quite successful: the glutamate receptor antagonists riluzole and gabapentin both were found to slow disease progression and prolong survival in SOD1^G93A^ mice, whereas vitamin E reportedly delayed onset of symptoms and slowed disease progression without prolonging survival [8]. Moreover, riluzole improved motor function as measured by spontaneous wheel-running activity and other motor assays [9]. These early findings in ALS mice actually followed, in time, the first human clinical reports that riluzole slowed SALS progression [14] and were thus actually rather confirmatory in nature rather than predictive. Four subsequent clinical trials noted a general positive effect of riluzole at 100 mg/day as indicated by quality of life measures such as tracheostomy-free survival, resulting in a consensus opinion that riluzole treatment added 2–4 months of life expectancy to ALS patients against the typical 4-5 year survival prognosis [15]. Currently, riluzole (Rilutek) is the sole drug approved by the United States Food and Drug Administration (FDA) for treatment of ALS.

Unfortunately, the early enthusiasm that mutant SOD1 transgenic mice would prove good predictors of clinical drug efficacy proved unfounded. First, the efficacy of riluzole, gabapentin, and vitamin E in murine ALS models proved very poorly reproducible [11]. Moreover, clinical trials of gabapentin [16], vitamin E [17], or the NMDA receptor antagonist memantine [18] failed to significantly benefit ALS patients though there were some nonsignificant trends noted in particular motor functional parameters such as rate of arm strength decline with gabapentin use [16]. In one study, vitamin E use combined with riluzole treatment may have tended to prolong time spent in earlier stages of ALS but had no effect on longer-term disease progression or survival in human ALS patients [17]. More recently, there has been some epidemiological evidence that long-term antioxidant supplementation with vitamin E may reduce risk for ALS [19] but in general the effects of both antioxidant and anti-excitotoxin therapies have been muted both in preclinical animal studies and actual human clinical trials.

The concept of anti-excitotoxins for ALS therapy is still a subject of active research and has yielded one very encouraging recent clinical result. The benzothiazole compound R-pramipexole (dexpramipexole or KNS-760704) emerged from Parkinson's disease research as a drug that could rescue dopaminergic neurons from glutamate excitotoxicity, but not by blocking Ca^2+^ influx; rather, the neuroprotective effects seemed mediated downstream from the glutamate-gated receptors and possibly map to action in the mitochondria [20]. Subsequent studies of dexpramipexole in ALS patients culminated in a recent report that 50–300 mg/day of the drug caused a dose-correlated positive change in functional decline as measured by a standard instrument, the ALS-Functional Rating Scale-Revised (ALSFRS-R) [21]. No other drug previously produced a significant effect on ALSFRS-R. Moreover, interim analysis indicated a near-significant trend (*P* < 0.07) toward increased lifespan in the ALS group receiving dexpramipexole [21]. Further clinical trials of dexpramipexole are ongoing.

## 3. Targeting Neuroinflammation by ****Suppressing Glial Activation and TNF*α* Production

Although the fatal risk in ALS is death of the motor neurons, it has become abundantly clear that this motor neuron death is not cell-autonomous but requires active contributions from surrounding nonneuronal cells (most likely, one or more glial cell types). The evidence for this is twofold. First, transgenic expression of mutant SOD1 restricted to neurons using a neurofilament light-chain promoter fails to produce motor neuron disease [22]. Second and even more convincingly, chimeric mice that express mutant SOD1 in both neurons and glia only develop disease when the mutant protein is heavily expressed in ambient nonneuronal cells, in which case the disease progression is correlated to the amount of glial mutant SOD1 expression [23]. Thus, it would seem that either astrocytes, microglia, or oligodendrocytes/Schwann cells (or some combination) contribute to motor neuron death in ALS.

Understanding the reasons for this could uncover feasible targets for pharmacological exploitation. As noted above, part of the glial involvement is passive, via loss-of-function(s); for instance, loss of EAAT2 expression in astrocytes likely exacerbates excitotoxic stress. A significant component of glial involvement in ALS is likely active, mediating motor neuron damage through active production of toxins or inappropriate release of paracrine factors. It has long been noted that microglial activation and proliferation occur in human and murine ALS [10, 24–26], suggesting that a neuroinflammatory process may be at play. This concept has gained credibility with the finding that particular cytokine and chemokine expression is perturbed in human ALS and animal models [27–33]. Most of the cytokine expression studies to date have been performed in SOD1 mutant animal models, simply because the central nervous system (CNS) tissue is easier to access at various stages of disease than is possible for human ALS where it is extremely difficult to assay neural parenchyma for inflammatory factors before (or even immediately following) death [10, 32–34]. Nonetheless, some studies have found elevated inflammatory cytokines in human ALS cerebrospinal fluid [27] or blood serum [27–31].

In particular, several studies have found that tumor necrosis factor-*α* (TNF*α*) is elevated in human ALS blood serum [30, 31]. In SOD1^G93A^ mice, TNF*α* message and protein are both elevated in spinal cord tissue, indicating endogenous production in CNS cells [32–34]. Moreover, TNF*α* is amongst the earliest and most aggressively upregulated gene products in SOD1^G93A^ mouse spinal cord tissue, becoming elevated before the appearance of obvious behavioral anomalies [32, 34]. Interestingly, cell culture studies of primary glia isolated from neonatal SOD1^G93A^ mice indicate that the cells exist in a metastable condition that is prone to fulminant activation. Such neonatal SOD1^G93A^ astrocytes hyperexpress endogenous TNF*α* as well as its principle receptor TNFRI in response to application of recombinant TNF*α* ligand or interferon-gamma (IFN*γ*) [35]. These same SOD1^G93A^ astroglia produce constitutively more prostaglandin-E2 (PGE_2_) and induce the leukotriene-producing arachidonate 5-lipoxygenase (5LOX) enzyme in response to inflammogens, to a much greater degree than do nontransgenic astrocytes or astrocytes overexpressing wild-type human SOD1 [35]. Similarly, primary SOD1^G93A^ microglia have been shown to superexpress TNF*α* in response to lipopolysaccharide (toll-like receptor ligand) stimulation *in vitro* [36]. A principle result of the hyperinflammatory cellular phenotype of SOD1^G93A^ glia is their enhanced production of reactive oxygen and nitrogen species and enhanced cytotoxicity [35, 36].

The association of TNF*α* and other inflammatory cytokines with toxic glial activation in ALS has encouraged a number of preclinical and clinical trials of therapeutics selected to either suppress TNF*α* production or otherwise blunt the neurotoxic activation of microglia. For instance, thalidomide derivatives which suppresses TNF*α* message translation in addition to other actions are amongst the more effective drugs tested in SOD1^G93A^ mice [10, 37, 38]. Unfortunately, human clinical trials of thalidomide for ALS have been unsuccessful due to lack of patient tolerance and excessive occurrence of sinus bradycardia [39, 40]. Doses of thalidomide that were tolerated by the ALS patients were insufficient to noticeably shift patient cytokine profiles [39]. It remains to be determined whether second-generation thalidomide derivatives such as lenalidomide, which are more active in SOD1^G93A^ mice [37, 38], prove safer and more effective than thalidomide against the human disease.

A surprisingly large number of compounds antagonize microglial activation by TNF*α* and other inflammogens *in vitro* [10, 41]. The list of such drugs includes cyclooxygenase inhibitors (e.g., classical nonsteroidal anti-inflammatory drugs or NSAIDS) and lipoxygenase inhibitors which apparently interfere with TNF*α* signaling inside microglia [41], as well as certain tetracyclic antibiotics exemplified by minocycline which act on mitogen activated protein kinase (MAPK) pathways to suppress toxic microglial activation and also stabilize mitochondrial [41, 42]. Unfortunately, to date, these indirectly acting anti-neuroinflammatory agents have not proven efficacious in the human clinic. There is no epidemiological or clinical evidence that NSAIDS generally protect against or slow SALS. Some clinical trials have been undertaken to test particular COX-II selective therapies for neurological diseases including ALS. For instance, the cycloogenase-2 inhibitor celecoxib, which decreased PGE_2_ and slowed progression of murine FALS [43], failed to have an effect in human trials [44]. Even more concerning, a formal clinical trial of minocycline actually caused acceleration of ALSFRS-R deterioration and produced neurological and gastrointestinal side-effects in the treatment group [45].

In more recent years, interest in TNF*α* pathways in ALS has waned somewhat, due largely to reports that crossing SOD1^G93A^ mice with TNF*α*-knockout mice did not significantly affect disease parameters [46]. There are however cogent reasons not to overinterpret this finding. TNF*α* is a pleiotropic cytokine that has both protective and toxic effects depending on the milieu in which it is expressed and whether one is speaking of soluble versus membrane-bound variants [47]; knocking out all TNF*α* constitutively would affect both aspects of cytokine function, possibly canceling out each, with little net effect on the ALS mouse. Also, one must consider the possible compensatory biology that would come to play during development of a complete TNF*α* knockout mouse. In any event, TNF*α* may be a useful model stimulus with which to probe both negative and positive aspects of neuroinflammation in ALS mice; the role of increased TNF*α* presence in human ALS remains subject to debate and further research.

Interest continues to identify safe anti-neuroinflammatory agents that might have some merit to test in ALS clinical trials. It is encouraging that polyphenols or similar compounds such as the 5LOX inhibitor nordihydroguaiaretic acid (NDGA) protect against murine ALS [41] and can be chronically tolerated, at least by healthy humans. In fact, NDGA was used as an antioxidant and anticaking agent in human food until the late 1950s [48]. NDGA is amongst the most potent tested inhibitor of TNF*α*-stimulated microglial activation *in vitro*, exceeding minocycline potency by 12-fold [41] extends lifespan in SOD1^G93A^ mice when administered orally beginning at symptomatic stages of disease [41] and also seems to promote glutamate uptake both *in vitro* in MN-1 cells and *in vivo* in spinal cord synaptosomes [49]. Interestingly, NDGA administered subcutaneously initially enhanced glutamate uptake in synaptosomes from SOD1^G93A^ mice but then lost efficacy with repeated administration apparently due to induced pharmacoresistance through upregulation of P-glycoprotein [49]. NDGA did not affect lifespan in this administration paradigm [47], in contrast to prior feeding paradigms [41], suggesting that precise dosage and route of administration may be important considerations.

## 4. Recent Initiatives to Support Neuron ****Structure and Function by Pharmacological Manipulation of Cytoskeletal Dynamics and Inter-Neuronal Mass Transport

Most ALS preclinical and clinical strategies thus far have fallen into two broad categories: preventing death of the motor neuron soma by pharmacologically targeting the neuron; or supporting motor neuron health indirectly by targeting the biology of ambient glia. Of course, minocycline garnered enthusiasm because of its apparent multiple targets of action both inside neurons and at the glial cell. Another tactic is gaining momentum in preclinical studies, which are beginning to reconceptualize ALS fundamentally as a cytoskeletal disease. This perspective is based on the fact that motor neuron cell death only happens near the end of an extensive clinical disease and thus likely is a final consequence of other neuronal processes. Indeed, ALS can be thought of as a progressive distal axonopathy [50, 51] beginning with motor end plate denervation and progressing through a period of axonal retraction through the ventral roots. In SOD1^G93A^ mice, motor end plates can be visualized along with their impinging axons [50, 51]. Beginning at 40–50 d, a full month before clinical symptoms or *α*-motor neuron death, the axons disappear from the motor end plate. This is followed by demonstrable shrinkage and loss of axons at the level of ventral routes and only finally by death of the attached cell bodies in the ventral horns [50, 51]. The denervation of motor end plates is probably cyclical, proceeding through a denervation-reinnervation process, because the affected muscles develop a characteristic fiber-type grouping that is commonly observed in situations where the distal motor nerve is injured. Compensatory axonal sprouting proximal to the muscle leads to clusters of grouped typeI (slow-fatigable, aerobic) and typeII (fast fatigable, anaerobic) fibers [50, 51]. Thus, if one could identify the initial triggers behind neuromuscular junction pathology in early ALS and/or the driving forces behind subsequent motor axon retraction, it might be possible to interfere to the net benefit of the motor neuron. 

Progress is being made toward both goals of identifying triggers and identifying driving processes for ALS axonopathy [52]. As for triggers, emphasis is becoming rather rapidly focused on axonal guidance or repulsion cues. Normally, during the course of embryonic development, neural crest cells are guided to migratory locations and growing axons find their appropriate targets through both negative and positive chemotactic cues. Most notably, semaphorins released by glial cells trigger a regulated process of axon growth cone collapse through receptor-mediated cytoskeletal reorganization [52]. As the actin microfilament network and tubulin-based microtubule tracts disintegrate on the side of the growth cone nearest the semaphorin source and extend on the further side, the axon bends away from the source of the repulsion cue. This guidance phenomenon may be of paramount importance in certain adult neuropathologies where the injured nervous system is attempting to heal itself by pruning and regrowing neural connections. In such circumstances, semaphorins and other paracrine factors become upregulated in order to sculpt new neural connections [52]. 

In 2007, Verhaagen's group published a curious observation that semaphorin 3A (Sema3A) is upregulated in terminal Schwann cells near the fast-fatigable fibers that are earliest to denervate in murine ALS [53]. To date, this observation has not been widely corroborated or built upon, but there are compelling reasons to do so when one considers the molecular mechanism of Sema3A action in relationship to our current knowledge of ALS axonopathy. The primary action of Sema3A is mediated by binding to receptor heterodimers composed of neuropilin-1 (NRP1) and plexin A [52, 54]. This event recruits intracellular kinases including Fyn kinase, triggering downstream activation of glycogen synthase kinase-3*β* (GSK3*β*) and cyclin-dependent kinase-5 (Cdk5) [52, 54]. GSK3*β* and Cdk5 act on a variety of microtubule-associated proteins including the collapsin response mediator protein (CRMP) system. CRMPs, particularly the CRMP2 isoform, function to stabilize both actin microfilaments and microtubules. Phosphorylation, expression downregulation, or sequestration into axonal aggregates can prohibit proper CRMP2 function and trigger severe axon retraction (reviewed in [54]). 

There are at least two additional pieces of evidence besides Verrhaagen's finding to suggest that the Sema3A-CRMP2-cytoskeleton signaling pathway is involved at least in murine ALS and potentially amenable to pharmacological intervention. First, Pettman and colleagues recently reported that a variant of CRMP4 is induced in cultured motor neurons by exposure to nitric oxide (^●^NO) [55]. Forced adenoassociated virus (AAV) mediated expression of CRMP4 in wild-type motoneurons triggered axon degeneration and cell death, whereas silencing of CRMP4 in mSOD1 motoneurons protected them from ^●^NO-induced death [55]. Thus, ectopic CRMP4 seems to oppose CRMP2 and promote neurodegeneration. If this is the case, then boosting CRMP2 function would be expected to compensate for CRMP4 in order to promote healthy neuritic structure and function.

Second, our group serendipitously discovered that a molecule called lanthionine ketimine (LK) binds CRMP2 to alter CRMP2 : tubulin interaction [56]. A cell-penetrating, synthetic LK-ester derivative (LKE) promotes differentiation of NSC-34 motor neuron-like cells, stimulating axonal extension, which also is seen in primary dorsal root ganglial neurons [54]. Curiously, LKE also potently suppresses TNF*α*-stimulated microglial ^●^NO production in culture which may or may not arise from the drug's apparent action upon CRMP2 [56]. Administration of LKE to SOD1^G93A^ mice beginning at symptomatic disease significantly slowed disease progression and extended lifespan [57]. We are currently exploring the development of LKE and related molecules as novel neurotrophic factors for ALS axonopathy and other indications, but this early data clearly suggests that (1) CRMP2-targeting signal transduction pathways are plausible candidates to explain the noncell autonomous, distal axonopathy component of ALS and (2) these signaling pathways are amenable to deliberate pharmacological intervention.

Other researchers have begun attempting more direct microtubule stabilization strategies in ALS mice, with some success. Notable is a 2007 study by Fanara et al. who reported that the microtubule stabilizing agent noscapine extended lifespan of SOD1^G93A^ mice by >10%, restored axonal transport deficits, and reduced motor neuron death [58]. This is encouraging when one considers that many attempts to prevent somatic death of motor neurons through antagonism of death pathways and apoptosis have generally met with poor results [10].

At this point, it is worth reconsidering the current list of generally accepted ALS genetic risk factors and Mendelian heritance factors (Table 1). At first glance, there appears to be little in the way of pathway overlap amongst these various genes. However, many of these gene products play roles in intracellular vesicle trafficking or axonal transport. Most of the remainder of the gene products can cause protein aggregation when mutated—thus, potentially creating impediments to cellular traffic along microtubule and microfilament tracks. Recent implications of axon guidance cue signaling and CRMP2 involvement in ALS dovetail remarkably well with the genetic epidemiology in suggesting that ALS is a syndrome driven in large part by defects in cytoskeleton-guided vesicle or axonal transport. If corroborated by future research, such hypothesis could form the basis for a unified theory of motor neuron disease and provide guidance for future drug discovery initiatives. 

## 5. Targeting Protein Aggregation and Oxidative Stress: Common Cell Biological Links

All of the targets mentioned thus far in this paper—excitotoxicity, neuroinflammation, glial activation, and protein cytoskeletal dysfunction—are not mutually exclusive but likely interact with one another quite dynamically and may be mechanistically united either through sharing common origins in protein aggregation disorders or common mechanisms of enhanced oxidative stress (reviewed in [59]). For instance, major proteins including mutant SOD1 and the tubulin-binding/stabilizing protein TCTP (translationally controlled tumor protein) are both excessively carbonylated in SOD1^ G93A^ spinal cord at latter stages of disease [59]. The protein degradation system component ubiquitin carboxy-terminal hydrolase-L1 and *α*, *β*-crystallin is also demonstrably hypercarbonylated in this mouse [59]. Thus, mutant SOD1 expression directly impinges on protein stability through the phenomena of (directly or indirectly) enhanced protein carbonylation; the oxidative effects upon protein degradation pathways might exacerbate. The oxidative stress to cytoskeletal proteins likely would decrease cells' ability to cope dynamically with stress, and posttranslationally modified protein aggregates likely could exacerbate neuroinflammatory microglial activation. The multitude of inter-related pathological stressors implicated in ALS mouse models may begin to explain why so many drugs produce small benefits, but so few drugs produce large benefits in this creature: there are many productive targets that are involved in disease progression, but suppressing one at a time is ineffective because the pathological current “flows around” every therapeutic barrier that the researcher erects.

## 6. Lessons from the Past, and Possibilities for the Future of ALS Therapy Development

The past two decades have witnessed a tremendous growth in knowledge about ALS. We now have a much more complete understanding of the genetic causes underlying FALS, though much less is known about risk factors for SALS. ALS transgenic models including mutant SOD1 transgenic mice but more recently including other transgenic constructs have provided powerful tools for exploring the basic biology of motor neuron disease and testing experimental therapeutics. This last point, however, is a pivot about which optimism for ALS therapy development has begun to vacillate. Numerous therapeutic strategies have produced small to moderate lifespan and functional effects in SOD1 mutant mouse studies (reviewed in [10, 11]), but the gains seemed capped at about 10% lifespan extension for reasons that still are not understood [10]. More troubling, efficacy effects in ALS transgenic mouse studies have been notoriously unreproducible [10, 11], and some of the more promising results have failed when translated to human clinical trials, or even worse, have caused harm to patients. What lessons can we learn from past translational efforts in ALS that might be applied productively to improve chances of future clinical trial success?

As others have discussed with great clarity [11], ALS mouse trials should be standardized along basic principles of randomization and observer blinding similar to the norm for human clinical trials. Furthermore attention should be placed to the dose and routes of administration in order to avoid true drug effects in mice that could never be safely recapitulated in humans. Drugs should be selected with respect to the timing of their likely mechanism of action: Drugs that target initial triggers of motor axonopathy may not be logical to test in humans beginning at mid-stage disease, though may be suitable for testing at early disease stages in FALS (however, limited patient availability is a hindrance for such trials); or early in SALS provided further improvements can be made in the speed of clinical diagnosis.

As for lessons regarding cellular targets for promising drug candidates, the only limited success to be achieved thus far in ALS clinical trials has stemmed from riluzole and tentatively, from dexpramipexole: two drugs that are thought to target either glutamate receptor activation or downstream consequences of glutamate excitotoxicity. Thus, priority might be given to drug candidates that have effects in cell or animal models that speak to excitotoxic pathways. On the other hand, not every antiexcitotoxin tested in human ALS or even in ALS mice can produce a benefit as exemplified by the failure of the NMDA receptor antagonist memantine [18]. From what we know of the genetic diversity of FALS combined with the diverse cellular pathway involvement in murine ALS pathogenesis, it would seem that drugs should be selected that target common downstream stress pathways that are activated during excitotoxicity as well as other forms of neurotoxic injury (e.g., oxidative insults or ligand-triggered axonal collapse pathways). On the other hand, caution is warranted when considering drugs that affect important cell physiological junctions such as mitochondrial biology, as such drugs necessarily carry a greater risk of unforeseen adverse events. This may be the case with minocycline, which apparently targets fundamental aspects of mitochondrial biology to the net benefit of ALS animal models but to the detriment of human ALS patients [45].

Continued effort to exploit particular neuroinflammatory pathways for ALS therapy seems warranted at this time, though researchers need to be cognizant that drug efficacy may vary from study to study in a capricious manner that is influenced in mice by pharmacotolerance in some cases (e.g., NDGA [49]). Finally, recent discoveries that axonal guidance and collapse pathways may be triggered near vulnerable ALS neuromuscular junctions and other serendipitous findings that the CRMP2/CRMP4 system is perturbed in ALS may point to an unexpected and exciting new concept in ALS therapy: rational pharmacological manipulation of cytoskeletal restructuring pathways to support the structure and function of motor axons.

Finally, it must be seriously considered that one or even two drugs will not treat ALS due to the multifactorial nature of the pathology. Though difficult to design and implement, trials of “cocktail” therapies consisting of multiple, rationally selected drug candidates may be worth attempting in future ALS mouse and human studies.

In summary, ALS therapy development efforts have been characterized by cycles of enthusiasm and deep disappointment, but scientific progress has been continual and may be nearing new milestones for clinical success if the initial clinical data regarding dexpramipexole effects on ALSFRS-R can be corroborated [21]. Rather than lament past failures, the ALS research community must carefully cultivate and renew its determination to understand and exploit novel, untested, but rational therapeutic targets. Such efforts may prove rewarding in the ALS clinic but in a broader sense may lead to new treatment modalities for other anterior horn diseases or wider classes of neurodegenerative pathologies.

## Figures and Tables

**Table 1:** Mendelian and non-Mendelian loci known to cause FALS or confer risk for SALS. Polymorphisms in the VEGF promoter that were originally associated with increased ALS risk have not been confirmed in subsequent studies but may act as modifiers of disease onset or progression in subsets of ALS cases [4].

Mendelian genes for heritable ALS (FALS)				
Gene	Location	Heritance	Protein	Pathway or effect
*ANG*	14q11.2	Dominant	Angiogenin	rRNA transcription
*ALS2*	2q33	Recessive	Alsin	Endosome/membrane trafficking
*C9ORF72*	9p21.2	Dominant	Uncharacterized	Altered C9ORF72 RNA splicing, formation of nuclear RNA foci
*FIG4*	6q21	Recessive	FIG4 homolog	Endosomal trafficking
*FUS*	16p11.2	Both	Fused in sarcoma	Altered RNA processing, formation of inclusion bodies
*OPTN*	10p13	Both	Optineurin	Golgi maintenance, membrane trafficking and exocytosis, formation of inclusion bodies
*SETX*	9q34.12	Dominant	Senataxin	DNA and RNA processing
*SOD1*	21q22.11	Almost always	Superoxide dismutase-1	Protein aggregation, possible gains of redox function, impaired axonal transport
*SPG11*	15q21.2	Recessive	Spatacsin	Impaired axonal transport
*TARDPB*	1p36.22	Dominant	TAR DNA binding	RNA processing, formation of protein inclusion bodies
*UBQLN2*	Xp11.231 dominant	X-linked	Ubiquilin-2	Proteasomal protein degradation, inclusion body formation
*VAPB*	20q13.32	Dominant	Vesicle-associated membrane protein VAMP	Vesicle trafficking
*VCP*	9p13.3	Dominant	Valosin-containing protein	Proteasomal degradation, endosomal trafficking, vesicle sorting

Susceptibility loci for sporadic ALS (SALS)				
Gene	Location	Polymorphism	Protein	OR (95% CI)

*GWA_9p21.2*	9p21.2	rs2814707	Unknown	1.25 (1.19–1.32)
*UNC13A*	19p13.1	rs12608932 homolog	Unc-13 Vesicle protein	1.18 (1.13–1.24)
*ATXN2*	12q24.12	Poly-Q	Ataxin-2	n.a.
