# Erratum to “Progress in Therapy Development for Amyotrophic Lateral Sclerosis”

**DOI:** 10.1155/2012/853030

**Published:** 2012-11-07

**Authors:** Kalina Venkova-Hristova, Alexandar Christov, Zarine Kamaluddin, Peter Kobalka, Kenneth Hensley

**Affiliations:** ^1^Department of Pathology, University of Toledo Medical Center, 3000 Arlington Avenue, Toledo, OH 43614, USA; ^2^Department of Neurosciences, University of Toledo Medical Center, 3000 Arlington Avenue, Toledo, OH 43614, USA

Please consider the following changes in Table 1.

## Figures and Tables

**Table 1:** Mendelian and non-Mendelian loci known to cause FALS or confer risk for SALS. Information in this table reproduces content in Tables  7 and 8 from Lill and Bertram, 2011 ([2], and databases therein). Polymorphisms in the VEGF promoter that were originally associated with increased ALS risk have not been confirmed in subsequent studies but may act as modifiers of disease onset or progression in subsets of ALS cases [58].

Mendelian genes for heritable ALS (FALS)				
Gene	Location	Heritance	Protein	Pathway or effect
*ANG*	14q11.2	Dominant	Angiogenin	rRNA transcription
*ALS2*	2q33	Recessive	Alsin	Endosome/membrane trafficking
*C9ORF72*	9p21.2	Dominant	Uncharacterized	Altered C9ORF72 RNA splicing, formation of nuclear RNA foci
*FIG4*	6q21	Recessive	FIG4 homolog	Endosomal trafficking
*FUS*	16p11.2	Both	Fused in sarcoma	Altered RNA processing, formation of inclusion bodies
*OPTN*	10p13	Both	Optineurin	Golgi maintenance, membrane trafficking and exocytosis, formation of inclusion bodies
*SETX*	9q34.12	Dominant	Senataxin	DNA and RNA processing
*SOD1*	21q22.11	Almost always	Superoxide dismutase-1	Protein aggregation, possible gains of redox function, impaired axonal transport
*SPG11*	15q21.2	Recessive	spatacsin	Impaired axonal transport
*TARDPB*	1p36.22	Dominant	TAR DNA binding	RNA processing, formation of protein inclusion bodies
*UBQLN2*	Xp11.231 dominant	X-linked	Ubiquilin-2	Proteosomal protein degradation, inclusion body formation
*VAPB*	20q13.32	Dominant	Vesicle-associated membrane protein VAMP	Vesicle trafficking
*VCP*	9p13.3	Dominant	Valosin-containing protein	Proteosomal degradation, endosomal trafficking, vesicle sorting
*PFN1*	17p13.3	Dominant	Profilin-1	Actin polymerization regulator

Susceptibility loci for sporadic ALS (SALS)				
Gene	Location	Polymorphism	Protein	OR (95% CI)

*GWA_9p21.2*	9p21.2	rs2814707	Unknown	1.25 (1.19–1.32)
*UNC13A*	19p13.1	rs12608932 homolog	Unc-13 vesicle protein	1.18 (1.13–1.24)
*ATXN2*	12q24.12	Poly-Q	Ataxin-2	N.a.

